# Organization of a Pediatric Scoliosis Surgery Task Force and Analysis of Clinical and Radiographic Outcomes

**DOI:** 10.1055/s-0044-1800946

**Published:** 2025-04-11

**Authors:** Carlos Augusto Belchior Bitencourt Júnior, Raphael de Rezende Pratali, Réjelos Charles Aguiar Lira, Sebastião Vieira de Morais, Anderson Matheus Medeiros de Araújo, Carlos Fernando Pereira da Silva Herrero

**Affiliations:** 1 Faculdade de Medicina de Ribeirão Preto da Universidade de São Paulo, Fortaleza, CE, Brasil; 2 Hospital do Servidor Público Estadual de São Paulo, São Paulo, SP, Brasil; 3 Faculdade de Medicina da Universidade Federal do Maranhão, São Luís, MA, Brasil

**Keywords:** scoliosis, spine diseases, surgery task force

## Abstract

**Objective** To demonstrate how a surgical task force can help provide access to surgical procedures for many patients waiting for surgery in the Brazilian Unified Health System (Sistema Único de Saúde - SUS, in Portuguese) waiting list.

**Methods** This is a retrospective cohort study involving 28 patients on the SUS waiting list included in a pediatric scoliosis surgery task force. We analyzed medical records, epidemiological data, and clinical and radiographic outcomes.

**Results** The data showed that the postoperative outcomes of curve correction and complications, such as infections, surgical wounds, pain, or other events, were consistent with the literature on the subject.

**Conclusion** Therefore, we believe a task force is critical for facilitating access to surgical procedures and restoring the quality of life of hundreds of patients.

## Introduction

Although scoliosis is defined as a spinal deformity in the coronal plane greater than 10°, it consists of a rotational vertebral deformity. 1 Idiopathic scoliosis is a structural disease in children at or near puberty .2 It can have different etiologies, including genetic syndromes, congenital spinal malformations, skeletal dysplasias, connective tissue conditions, and neuromuscular diseases. 1

Most cases present a low curvature magnitude per the Cobb angle, 3 with no significant clinical repercussions. The prevalence of curvatures with a magnitude large enough to consider surgical treatment is extremely low, ranging from 0.04 to 0.4%. 1 4

Most scoliosis cases present non-progressive deformity and do not require surgical treatment. However, some cases present rapid curvature progression requiring surgery due to the complex deformities associated with pediatric scoliosis, leading to the idea of organizing surgical task forces. 5

Tasks forces aim to speed up the processing of elective surgeries. Regarding the topic addressed in the present study, the Scoliosis Research Society (SRS) pioneered the development of programs to organize training actions for reference centers in the surgical treatment of pediatric scoliosis, culminating in surgical task forces, as recently described in the literature. 6 7

In the current paper, we describe a task force for scoliosis treatment and its epidemiological, logistical, radiographic, and clinical data to encourage similar initiatives.

## Materials and Methods

This study was retrospective, with an observational cohort. Participants were selected from the Brazilian Unified Health System's (Sistema Único de Saúde [SUS], in Portuguese) waiting list in Maranhão, Brazil, who underwent outpatient reassessment by the group and outpatient anesthesia evaluation. The exclusion criteria were previous surgeries, active infection, and lack of anesthesia for surgery.

We informed all selected patients about the study and invited them to participate after signing the informed consent form and the underage assent form approved by the research ethics committee. The task force occurred at Hospital Universitário from Universidade Federal do Maranhão (HU-UFMA). Twenty-eight patients who underwent surgical procedures between February 1 and 4, 2021, participated in the study.

We collected and recorded demographic data, including gender, age, weight, etiology of the deformity, and time between inclusion on the waiting list and surgery. We calculated the mean, median, and standard deviation values for variables and the frequency of each scoliosis etiology.

We evaluated and tabulated surgical data, including intraoperative bleeding, neurophysiological changes, implant type, and number of implants. In addition, we assessed drainage volume, blood transfusions, and the presence or absence of infections in the postoperative period.

We determined the deformity magnitude per the Cobb angle from all patients in the preoperative and immediate postoperative periods using the SurgiMap (Nemaris Inc., Methuen, MA, USA) application.

Moreover, we collected data from the professionals involved in the task force, such as their area of medical specialty and the Brazilian region in which they operate, through interviews. We recorded logistical data, including the number of operating rooms and intensive care unit (ICU) bed reserves as well as specific needs subjectively observed at each process stage.

## Results

### Demographic and Preoperative Clinical Data

The participants were 10 to 17 years old, including 24 were females and 4 males. Idiopathic (childhood, adolescent, and juvenile) scoliosis was the most common condition, (observed in 20 subjects; 71%), followed by congenital (4 patients; 14%), neuromuscular (3 subjects; 11%), and diplomyelia (1 patient; 4%) etiologies ( Table 1 ).

**Table 1:** Sociodemographic data

Patient	Scoliosis etiology	Pre-procedural height (m)	Weight (kg)	Gender
M1	Adolescent idiopathic	1.65	46	Female
M2	Neuromuscular	1.43	30	Female
M3	Congenital	1.39	35	Female
M4	Adolescent idiopathic	1.56	48	Female
M5	Adolescent idiopathic	1.54	46	Female
M6	Adolescent idiopathic	1.57	44	Female
M7	Child idiopathic	1.48	60	Female
M8	Adolescent idiopathic	1.62	43	Female
M9	Diplomyelia	1.35	28	Female
M10	Adolescent idiopathic	1.66	55	Female
M11	Adolescent idiopathic	1.55	54	Female
M12	Juvenile idiopathic	1.72	59	Female
M13	Child idiopathic	1.59	43	Female
M14	Adolescent idiopathic	1.65	52.5	Female
M15	Adolescent idiopathic	1.59	47	Female
M16	Neuromuscular	1.30	30	Male
M17	Juvenile idiopathic	1.42	41	Male
M18	Congenital	1.64	60	Male
M19	Juvenile idiopathic	1.53	37.2	Male
M20	Adolescent idiopathic	1.61	47	Female
M21	Neuromuscular	1.30	22	Female
M22	Congenital	1.61	46	Female
M23	Juvenile idiopathic	1.68	55	Male
M24	Adolescent idiopathic	1.66	50	Female
M25	Adolescent idiopathic	1.62	56	Female
M26	Congenital	1.27	23	Female
M27	Adolescent idiopathic	1.64	46	Female
M28	Adolescent idiopathic	1.52	38	Female

Note: Specification of scoliosis type, height, weight, and gender.

Source: Authors (2023).

The mean age at diagnosis/surgical indication was 10.2 years; the age at surgery was 15.1 years, and the mean time from the diagnosis to the procedure was 4.7 years.

### Intraoperative Results

Regarding surgical data ( Table 2 ), the mean blood loss was 768.61 ml, and 3 patients required blood transfusion. The mean surgical time was 200.74 minutes. Five subjects presented transient neurophysiological changes but no postoperative neurological deficits.

**Table 2:** Surgical data

Patient	Blood loss (mL)	Neurophysiological abnormality	Episode description	Techniques	Proximal level included in arthrodesis	Distal level included in arthrodesis	Surgical time (min)
M1	790	Yes	Motor potential drop at derotation. Signals normalized after bar removal. Surgery was completed without further complications.	NA	T8	L3	180
M2	870	No	NA	Intraoperative traction: bipolar	T1	Ilium	310
M3	110	No	NA	Laminectomy; transforaminal lumbar interbody fusion	L4	S1	180
M4	580	No	NA	NA	T4	L2	200
M5	1115	No	NA	NA	T4	T12	180
M6	470	Yes	Motor potential drop on the left side. The potential returned after mean blood pressure and room temperature increase.	Intraoperative traction	T4	L1	220
M7	970	Yes	Potential drop on the left side with normalization after traction removal	Intraoperative traction.	T2	L2	180
M8	650	No	NA	NA	T11	L3	100
M9	1140	No	NA	Intraoperative traction; costoplasty	T3	L3	240
M10	550	No	NA	Intraoperative traction	T4	L3	300
M11	960	No	NA	NA	T4	L4	200
M12	1400	No	NA	Osteotomies (3)	T4	L4	300
M13	1010	No	NA	Intraoperative traction	T4	L2	200
M14	610	No	NA	NA	T4	T12	145
M15	900	No	NA	NA	T6	L3	135
M16	350	No	NA	Intraoperative traction; bipolar	T1	Ilium	250
M17	1500	No	NA	Intraoperative traction; osteotomies (3)	T2	L2	220
M18	560	No	NA	Osteotomies (3)	T2	L2	280
M19	980	No	NA	Osteotomies (3); intraoperative traction	T4	L3	120
M20	690	No	NA	NA	T10	L4	120
M21	840	No	NA	Intraoperative traction; bipolar	T1	Ilium	220
M22	1,250	Yes	Motor and sensory potential drop during osteotomy. The potential normalized after decompression and osteotomy completion.	Asymmetric pedicle subtraction osteotomy in T10	T6	L3	270
M23	740	No	NA	NA	T3	T12	200
M24	950	No	NA	Osteotomies (3)	T3	L2	200
M25	300	No	NA	NA	T4	T12	100
M26	330	Yes	Potential drop in the left leg with normalization after traction decrease	intraoperative traction	C7	L1	240
M27	820	No	NA	Proximal level translation	T4	L3	200
M28	590	No	NA	NA	T11	L4	150

Note: Specification of intraoperative bleeding, neurophysiological abnormalities, procedures performed, arthrodesis levels, and surgical time.

Source: Authors (2023).

Seventeen patients underwent traction procedures and type 2 osteotomies; five, intraoperative traction; three, intraoperative traction combined with the bipolar technique; three, intraoperative traction and osteotomies; one, intraoperative traction combined with costoplasty; and three patients underwent osteotomies alone.

Other surgical procedures included asymmetric pedicle subtraction osteotomy (PSO) of T10, laminectomy, and transforaminal lumbar interbody fusion (TLIF).

Regarding arthrodesis, the proximal level of screw insertion was the thoracic region, mainly in the T4 vertebra; in 1 patient, it occurred in the cervical region (C7) and, in another subject, in the lumbar region (L4). The distal level concentrated in the lumbar region; in four patients, it occurred in the thoracic region (T12), in the sacrum region (S1) in one patient, and, in three subjects, the procedure occurred in the hip region (ilium).

All patients received implants. In total, we used 457 implants, including screws, rods, hooks, and sublaminar bands.

### Clinical Postoperative Outcomes and Complications

The clinical postoperative data ( Table 3 ) show that the average time to discharge was 6.92 days. All patients presented secretions or blood in the drain on the 1 ^st^ day after surgery (average volume, 370.05 mL), 27 (96%) subjects on the 2 ^nd^ day (average volume, 252.59 mL), 9 (32%) on the 3 ^rd^ day (average volume, 144.66 mL), and only 1 (4%) on the 4 ^th^ day (280 mL).

**Table 3:** Postoperative outcomes

Patient	Time to discharge	Right drain 1 output (mL)	Right drain 2 output (mL)	Right drain 3 output (mL)	Right drain 4 output (mL)	Transfused blood bags	Number	Hemoglobin, PO1	Hemoglobin, PO3	Infections	Infection description	Other complications	Complication description
M1	5 days	225	175	150		Yes	2	8.2	9.8	No		Yes	Nausea and vomiting
M2	12 days	680	350			Yes	3	12.6	12.8	No		No	
M3	4 days	300	200			No		11.1	11.0	No		No	
M4	4 days	370	250			No		10.6	10.1	No		No	
M5	6 days	317	150			No		10.2	9.9	No		No	
M6	8 days	454	300			No		9.4	9.1	No		No	
M7	7 days	144	180			Yes	2	8.2	10.2	No		Yes	Nausea and vomiting
M8	5 days	300	150	86		No		9.0	8.1	No		Yes	Nausea and vomiting
M9	5 days	480	200			Yes	2	8.1	12.2	No		Yes	Intense pain
M10	6 days	290	278			No		10.1	10.7	No		Yes	Dyspnea at moderate exertion
M11	6 days	450	140			Yes	4	8.4	12.3	No		Yes	Nausea and vomiting
M12	18 days	250	500	500		Yes	3	7.5	9.3	Yes	Surgical site infection with *Klebsiella pneumoniae*	Yes	Secretive surgical wound, debridement on Feb 12
M13	7 days	155	45			No		9.7	8.1	No		Yes	Nausea and vomiting
M14	6 days	300	188			Yes	1	8.5	10.7	No		Yes	Moderate pain
M15	5 days	390	380	120		No		9.5	9.6	No		No	
M16	14 days	630	450			No		11.2	10.3	Yes	UTI by *Enterobacter cloacae*	Yes	Sacral stasis ulcer
M17	5 days	550	190			Yes	3	7.6	10.5	No		Yes	Metabolic acidosis
M18	5 days	250	450	53		No		13.6	11.3	No		No	
M19	6 days	500	150			Yes	1	9.7	11.0	No		Yes	Metabolic acidosis
M20	18 days	370	500	115	280	Yes	2	9.1	9.4	No		Yes	Functional obstruction, seizures, hypokalemia.
M21	8 days	400	344			No		10.9	11.1	No		No	
M22	5 days	725	400	118		No		9.0	8.4	No		Yes	Intense pain
M23	5 days	400	200			No		11.4	11.0	No		No	
M24	6 days	303	100			No		9.4	8.4	No		Yes	Bladder globe, metabolic acidosis
M25	4 days	350	170	100		No		8.9	8.9	No		No	
M26	5 days	358	180			Yes	2	8.0	11.2	No		No	
M27	5 days	200				Yes	2	8.0	10.9	No		Yes	Nausea and vomiting
M28	4 days	220	200	60		No		10.3	10.4	No		No	

Abbreviations: PO, postoperative day; UTI, urinary tract infection.

Note: Specification of postoperative variables and complications.

Source: Authors (2023).

Twelve patients (43%) required blood bags postoperatively. Six received two, three received three, two received one, and one received four bags.

Among complications, two subjects presented infection; one had urinary tract infection (UTI) by *Enterobacter cloacae* , and the other had a surgical site infection by *Klebsiella pneumoniae* . In percentage terms, by etiology, we observed 5% of surgical site infections in idiopathic cases, with no other surgical site infections. Sixteen patients (57%) presented some complication, including eight with nausea and vomiting, two with intense pain, one with moderate pain, two with metabolic acidosis, one with acidosis and urinary alterations (bladder globe), one with a sacral stasis ulcer, one required surgical debridement, one presented dyspnea on moderate exertion, and one presented functional obstruction, seizures, and hypokalemia.

### Pre- and Postoperative Radiographic Results

Regarding the radiographic data, the preoperative mean Cobb angles were 29.3 degrees (range, 0–68) for the upper thoracic curve, 3.6 degrees (range, 0–120) for the lower thoracic curve, 42.2 degrees (range, 16–79) for the lumbar curve, 36.6 degrees (range, 5 to 75) of thoracic kyphosis, and 55.6 degrees (range, 8–86) of lumbar lordosis ( Table 4 ).

**Table 4:** Radiographic outcomes

Patient	Preoperative proximal thoracic Cobb angle (degrees)	Preoperative distal thoracic Cobb angle (degrees)	Preoperative lumbar Cobb angle (degrees)	Preoperative kyphosis Cobb angle (degrees)	Preoperative lordosis Cobb angle (degrees)	Postoperative proximal thoracic Cobb angle (degrees)	Postoperative distal thoracic Cobb angle (degrees)	Postoperative lumbar Cobb angle (degrees)	Postoperative kyphosis Cobb angle (degrees)	Postoperative lordosis Cobb angle (degrees)
M1	22	49	35	43	60	20	29	21	2	36
M2	50	86	36	17	46	34	61	25	4	56
M3	0	0	55	38	66	0	0	50	38	46
M4	35	72	38	16	58	16	23	9	27	47
M5	30	48	36	27	57	3	27	31	20	50
M6	33	106	55	47	50	22	29	34	36	45
M7	49	83	44	58	86	38	44	25	49	68
M8	15	36	45	5	30	24	33	16	13	34
M9	68	120	20	75	56	20	56	0	40	52
M10	27	56	68	5	48	5	23	48	33	45
M11	66	103	40	41	55	56	54	21	15	49
M12	0	50	79	5	8	0	26	23	19	33
M13	49	78	43	28	57	35	44	20	39	55
M14	9	54	40	18	65	15	25	7	32	20
M15	16	49	48	38	71	15	37	31	30	52
M16	12	22	50	28	72	20	14	34	28	80
M17	30	97	37	70	45	27	65	25	50	50
M18	40	74	47	56	58	33	54	27	41	36
M19	30	94	38	58	53	35	38	17	38	40
M20	6	36	47	36	52	6	10	20	40	50
M21	45	101	16	62	68	40	78	2	3	45
M22	51	89	44	70	76	28	53	21	55	55
M23	23	60	34	25	43	26	30	26	28	35
M24	43	57	30	42	56	29	35	16	38	50
M25	19	40	20	10	71	5	0	0	12	60
M26	45	87	31	65	57	37	48	10	33	24
M27	6	0	40	23	48	0	0	16	40	40
M28	2	34	66	19	45	0	24	27	30	37
	29.32142857	63.60714286	42.21428571	36.60714286	55.60714286	21.03571429	34.28571429	21.5	29.75	46.55555556
Percentage of curve correction			High thoracic: 28%	Low thoracic: 47%	Lumbar: 50%					

Note: Radiographic results calculated by the Cobb method pre and postoperatively.

Source: Authors (2023).

In the immediate postoperative period, the average correction was 28% for the upper thoracic curve, 47% for the lower thoracic curve, and 50% for the lumbar curve (postoperative mean values of 21 degrees, 34.2 degrees, and 21.5 degrees, respectively).

Addressing non-idiopathic scoliosis separately, the correction percentage of the main curve was 28% in neuromuscular scoliosis and 30% in congenital scoliosis.

### Organizational and logistical results

The hospital structure included an outpatient clinic, a radiology department, 5 dedicated surgical rooms available for 4 days, and approximately 10 to 15 ICU beds for routine postoperative care. The surgical team included volunteer doctors and doctors from the hospital staff. The implants were donated, so we could not assess their costs with precision.

**Fig. 1:**
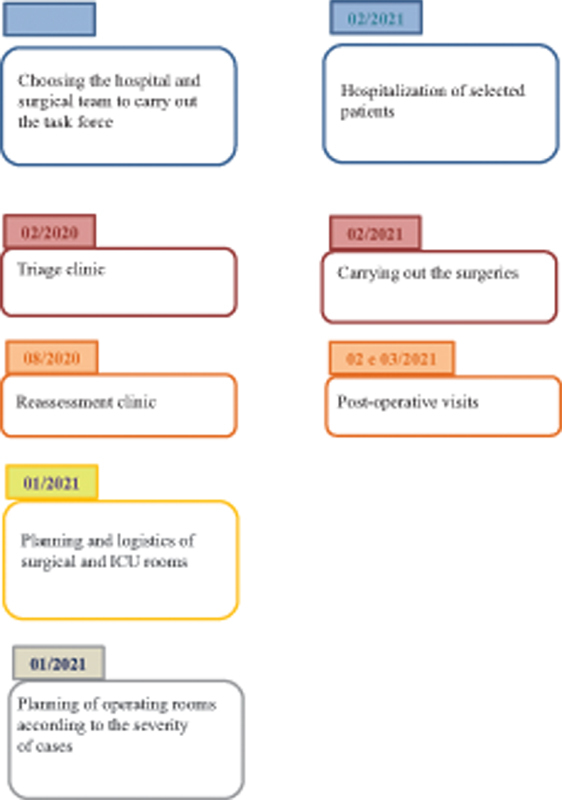
Task force schedule.

Although the surgeries occurred over 4 days in February 2021, the task force organization began in 2020 ( Fig. 1 ).

## Discussion

Most patients were female, including 24 (86%) of the 28 subjects. This data is consistent with another study with 169 patients, including 121 (71.6%) females. 7 Another study on the incidence of adolescent idiopathic scoliosis (AIS) in several countries noted that the prevalence and severity of scoliosis were higher in girls. 8

The present study also found a higher prevalence of idiopathic scoliosis, accounting for 20 cases (71%). In the current literature, the overall prevalence of AIS ranges from 0.47 to 5.2%. 8 A review from Pereira and Gomes 9 mentions studies corroborating our results: in a sample of 358 subjects, 16 had AIS (prevalence, 4.8%); 10 in a sample of 3,105 subjects, 38 had AIS (prevalence, 5.3%); 11 among 418 subjects, 18 adolescents had AIS (prevalence, 4.3%); 12 among 2,562 adolescents, 37 had AIS (prevalence, 1.5%). 13 These data demonstrate that our study is consistent with the epidemiological literature.

Regarding postoperative complications, discomforts are frequent, including nausea and vomiting in the first hours after surgery due to oral refeeding or anesthesia, dyspnea, oliguria, 14 odynophagia, pain at the intravenous injection sites, insomnia, and constipation. 15 16 Eight patients presented nausea and vomiting, one had dyspnea with moderate exertion, and one had functional obstruction; these are common reactions in postoperative patients.

As for pain, a citation highlights that “[...] the surgical wound is not spontaneously painful after 48 hours of the surgical procedure ”14 . Therefore, it is critical to ascertain the pain level and perform the required procedures. Among the 28 patients, only 2 presented intense pain, while 1 had moderate pain, with no other complications.

Other more painful occurrences may occur in the postoperative period of surgical procedures in general, including bleeding, wound infection, venous thrombosis, respiratory failure, pulmonary thromboembolism, pulmonary atelectasis, and UTI. 15 16

Other studies on postoperative infections in patients treated for spinal deformities report UTIs, sphincter control loss, contamination, wound infections, gastrointestinal disorders, and pulmonary complications .17 They also reported a higher infection risk in patients with neuromuscular scoliosis than those with AIS. In the present study, complications and infections were common in postoperative patients .18

Sensitivity loss in the extremities may cause loss of bowel or bladder control, especially in patients with neuromuscular scoliosis. 18 The complications observed in patient M16 (with neuromuscular scoliosis) may be related to the scoliosis type as they could not walk and had a UTI and a sacral ulcer.

The most severe complication occurred in patient M12 (AIS), who developed a surgical wound infection, a complication also observed in other studies. 15 16 17 18 The treatment was surgical debridement mentioned as the usual therapy, 18 with surgical site irrigation. In the literature, the infection rate in surgery for AIS ranged from 0.9 to 3%, and, for neuromuscular scoliosis, it ranged from 4.2 to 20%. In our study, the prevalence of surgical wound infection was approximately 3.5% in the general analysis and 5% among idiopathic cases.

Regarding other complications, the data depends on the consulted databases, ranging from 5 to 23% in AIS. More recent data from the SRS database, from 2011, cited a complication rate of 6.3% for all cases of idiopathic scoliosis (IS). In our study, 16 patients (57%) presented complications; those associated with the specific type of surgery affected 6 out of the 28 patients, that is, a prevalence rate of 21%, with only one major complication (which led to reoperation), a surgical site infection.

Since the complication and infection rates are consistent with the literature, the short time of surgery has no relationship with complications.

Radiographically, the overall average correction was 28% for the high thoracic curve, 47% for the low thoracic curve, and 50% for the lumbar curve (postoperative averages of 21 degrees, 34.2 degrees, and 21.5 degrees, respectively). After stratification by non-idiopathic etiologies, the main curve correction was 28% in neuromuscular scoliosis and 30% in congenital scoliosis. In a study 19 analyzing several postoperative outcomes, an article published in 1973, with 71 participants using Harrington rods, Risser plaster, and early ambulation, reported a mean preoperative curve of 56°, with 54% correction on the day of surgery and 46% correction at follow-up. In 1989, a study with 352 patients undergoing posterior spinal fusion reported a mean preoperative curve of 54° and a mean correction of preoperative active supine tilt of 48%. The average correction at surgery was 52% and 40% at the 2-year follow-up .19

In 2004, a comparative study of 4 different instrumentations (double rod, multi-hook systems) involving 127 patients and using the C-D Horizon, Moss-Miami, TSRH, and Isola systems showed an average correction of 63% for the C-D Horizon and Moss-Miami and 58% for the TSRH and Isola. 19

**Fig. 2:**
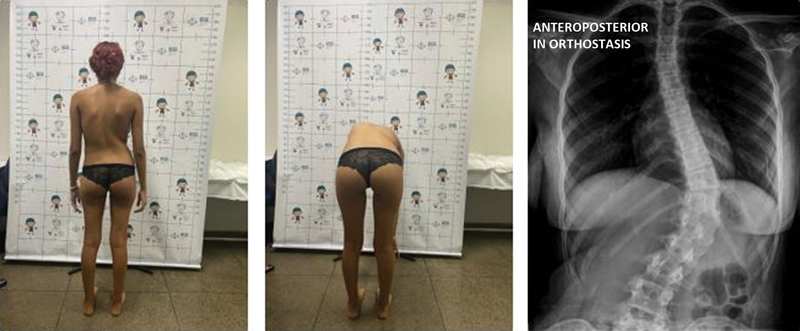
Preoperative image of patient M21. Source: Authors (2023).

The curve correction over the years remained similar, and the values achieved in our study are consistent with the literature. As such, although the curve correction was not complete, the outcomes were satisfactory. 19 Figs. 2 3 4 5 visually demonstrate the correction level achieved in some patients.

**Fig. 3:**
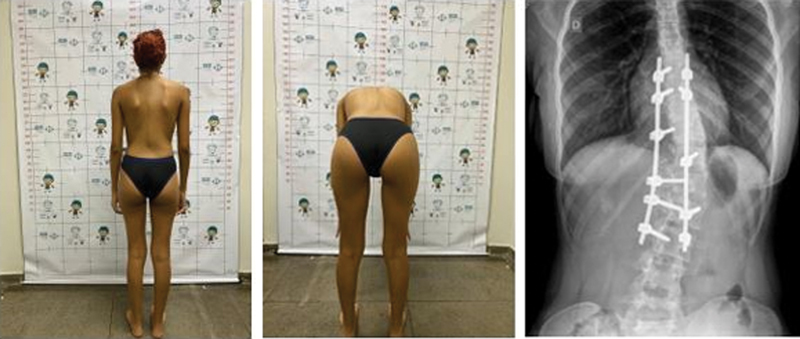
Postoperative outcomes in patient M1. Source: Authors (2023).

**Fig. 4:**
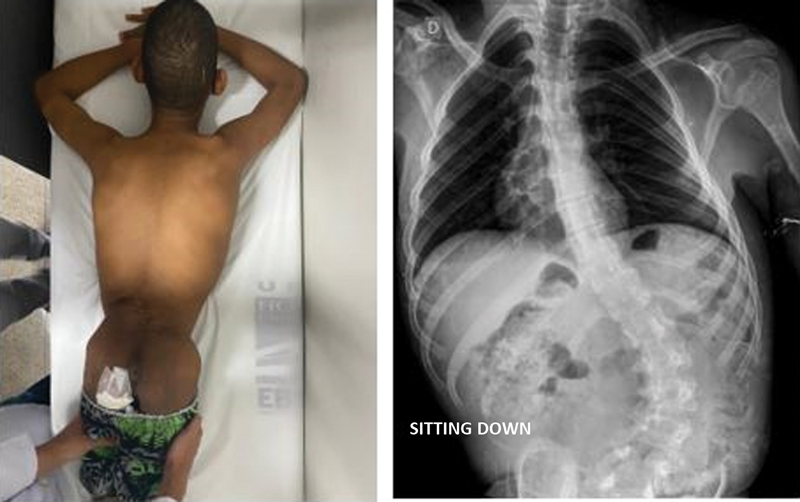
Preoperative image of patient M16. Source: Authors (2023).

**Fig. 5:**
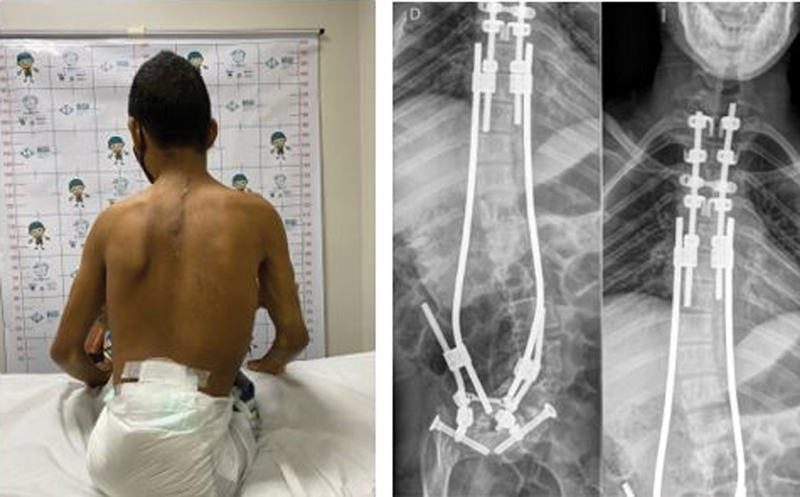
Postoperative outcomes in patient M16. Source: Authors (2023).

Regarding logistics, we had some difficulties during the task force, but no similar studies addressed them. In the preoperative period, we faced challenges in publicizing the triage clinic and contacting several patients. In addition, we needed to train radiology technicians to perform spinal panoramic radiographs in orthostasis. We also required large treatment rooms to take photographs and clinically evaluate the patients in the triage clinic. For the preoperative evaluation, we needed an anesthesiology clinic to assess and prepare patients for the procedure.

The logistical difficulties during surgery included gathering staff and resources for the stipulated task force time. The task force required a team of professionals from various areas of expertise and different Brazilian states with experience in scoliosis surgery and availability.

In the postoperative period, the challenges included ICU room availability. We required approximately 10 to 15 beds at the same time because sometimes more severe patients could not be discharged from the ICU on the first postoperative day. In addition, we needed nursing and physical therapy teams trained in scoliosis treatment procedures to maintain drains, change dressings, or ensure early ambulation.

The literature about surgical task forces 5 7 for correcting scoliosis curves provided no data on logistical difficulties or reported complications potentially warranted by these joint efforts. However, some news reports provided data on task forces and highlighted critical points.

The Regional Medical Council of the State of Bahia (CREMEB, for its acronym in Portuguese), 20 in 2018, warned about some issues in task forces, including problems resulting from the scenarios in which these surgeries occur and complication risks from surgical procedures, especially because of the potential lack of qualified personnel for intraoperative and postoperative monitoring.

Despite the news reports on complications in task forces, none deals with scoliosis surgery, and the cases with problems are low compared to the number of benefited people.

In December 2022, Centro Estadual de Reabilitação e Readaptação Dr. Henrique Santillo (CRER), a rehabilitation center from the Health Department of Goiás, Brazil, performed elective scoliosis surgeries in patients on the SUS list. Twenty patients underwent treatment; some had been on the waiting list for about 5 years, and the surgery improved their quality of life. 21

In Pernambuco, Brazil, the traumatology and orthopedics team at Hospital Otávio de Freitas performed a surgical series for scoliosis treatment in 18 patients to minimize the SUS waiting list. This team did 4 procedures per day in 3 dedicated surgical rooms and used 16 beds from the adult and pediatric wards, trauma surgical center, ICU, and recovery room. 22

In our study, the average age at diagnosis was 10.2 years, and surgery occurred at 15.1 years old, with a waiting time for the procedure of 4.7 years. In Brazil, a study 23 with 51 patients, all diagnosed from ages 10 to 17 years old, and the average waiting time for surgery was 25.41 months (ranging from 2–180 months). However, some patients waited for the surgery for up to 15 years. This waiting time can compromise the patient's quality of life, self-image, satisfaction, and functionality. 24

We did not analyze the quality of life or personal satisfaction questionnaires because of logistical issues.

## Conclusion

Despite the difficulties in organizing similar actions and some complications, it seems feasible to encourage the multiplication of these task forces in more hospitals due to the high number of patients on waiting lists for scoliosis surgery. However, it is fundamental to emphasize the need for more actions using this model to assess accurately its safety and applicability, especially in severe and non-idiopathic cases.
