# Supplementary material for: Organization of a Pediatric Scoliosis Surgery Task Force and Analysis of Clinical and Radiographic Outcomes
Source: Rev Bras Ortop (Sao Paulo). 2025 Apr 11;60(1):s00441800946. doi: 10.1055/s-0044-1800946 (PMC12020556; doi:10.1055/s-0044-1800946)
Supplement: Related texts

# Research article: Organização de um mutirão de cirurgia de escoliose pediátrica e análise dos resultados clínicos e radiográficos

**Keywords:** doenças da coluna vertebral, escoliose, mutirão de cirurgia

## Abstract

**Objetivo** Mostrar como o mutirão de cirurgias pode auxiliar no acesso ao procedimento cirúrgico para muitos pacientes que aguardam para realizar cirurgia na fila de espera do Sistema Único de Saúde (SUS).

**Métodos** Trata-se de um estudo retrospectivo de coorte que contou com 28 pacientes da lista de espera do SUS, incluídos em um mutirão de cirurgia de escoliose pediátrica, no qual foram realizadas revisão de prontuário, análise epidemiológica e de resultados clínicos e radiográficos.

**Resultados** Os dados mostraram que os resultados pós-cirúrgicos de correção das curvas e o nível de complicações observadas nos pacientes, tais como o acometimento de infecções, feridas cirúrgicas, dor ou outros tipos de eventos, são compatíveis com a literatura sobre tema.

**Conclusão** Sendo assim, entende-se que o mutirão é importante para facilitar o acesso ao procedimento cirúrgico e devolver a qualidade de vida a centenas de pacientes.

## Introdução

A escoliose consiste em um termo utilizado para definir uma deformidade da coluna vertebral no plano coronal superior a 10°, embora consista em uma deformidade vertebral rotacional. 1 A escoliose idiopática é uma doença estrutural, que acomete crianças na puberdade ou perto dela. 2 Pode se originar a partir de diferentes etiologias, associada a síndromes genéticas, malformações congênitas da coluna vertebral, displasias esqueléticas, doenças do tecido conjuntivo e neuromusculares. 1

A grande maioria desses casos apresenta baixa magnitude da curvatura, medida pelo ângulo de Cobb, 3 o que não traz grandes repercussões clínicas. A prevalência de curvaturas com magnitude grande o suficiente para considerar tratamento cirúrgico é extremamente baixa, entre 0,04 e 0,4%. 1 4

A maioria dos casos de escoliose apresentam deformidade não-progressiva e não requerem tratamento cirúrgico. No entanto, alguns casos apresentam progressão rápida da magnitude da curvatura, necessitando de tratamento cirúrgico por conta das deformidades complexas associadas à escoliose pediátrica, o que levou à ideia da organização de projetos para a realização de mutirões de cirurgia. 5

Os mutirões visam dar celeridade ao andamento das filas de cirurgias eletivas. Com relação à temática abordada neste estudo, a *Scoliosis Research Society* (SRS) foi pioneira no desenvolvimento de programas para a organização de ações de treinamento de centros referência no tratamento cirúrgico de escoliose pediátrica, culminando com a realização de mutirões de cirurgia, o que foi recentemente descrito na literatura. 6 7

Neste trabalho, descreveremos em seus pormenores um mutirão de tratamento de escoliose, com dados epidemiológicos, logísticos, radiográficos e clínicos, como modo de incentivo a iniciativas similiares.

## Materiais e Métodos

Trata-se de um estudo observacional retrospectivo de coorte. Os participantes foram selecionados da lista de espera do Sistema Único de Saúde (SUS) do estado do Maranhão e passaram pelo ambulatório de reavaliação com o grupo e pelo ambulatório de avaliação anestésica, tendo como critérios de exclusão: cirurgias prévias, infecção ativa e falta de condição anestésica para o procedimento.

Todos os pacientes selecionados foram orientados sobre o estudo e convidados a participarem, mediante assinatura do termo de consentimento livre e esclarecido e do termo de assentimento do menor, após aprovação pelo comitê de ética em pesquisa. O mutirão aconteceu no Hospital Universitário da Universidade Federal do Maranhão (HU-UFMA). Participaram 28 pacientes submetidos a procedimentos cirúrgicos no período de 1 a 4 de fevereiro de 2021.

Os dados demográficos foram colhidos e registrados, incluindo sexo, idade, peso, etiologia da deformidade, e tempo de espera entre a inclusão na lista de espera até a cirurgia, tendo sido calculados média, mediana e desvios padrões das variáveis, assim como a frequência de cada etiologia de escoliose.

Foram avaliados e tabulados os dados relacionados ao procedimento cirúrgico em si, como sangramento intra-operatório, alterações neurofisiológicas, quantidade e tipos de implantes utilizados, dentre outros fatores. Também foi avaliado, no pós-operatório dos pacientes, dados sobre o dreno, transfusões sanguíneas e a presença ou não de infecções.

A magnitude da deformidade, determinada pelo ângulo de Cobb foi calculada para todos os pacientes no pré e no pós-operatório imediato. Utilizou-se o aplicativo SurgiMap (Nemaris Inc., Methuen, MA, EUA) para realização dos cálculos.

Também, foram coletados dados dos profissionais envolvidos no mutirão, como área de especialidade na medicina e região da federação de atuação, através de entrevistas. Foram contabilizados aspectos organizacionais, como a quantidade de salas cirúrgicas, reservas de leitos de unidade de terapia intensiva (UTI), bem como aferidas as necessidades específicas observadas subjetivamente em cada etapa do processo.

## Resultados

## Resultados Demográficos e Pré-operatórios Clínicos

Os participantes tinham entre 10 e 17 anos, incluindo 24 do sexo feminino e 4 do sexo masculino. Observou-se maior incidência da escoliose idiopática (infantil, adolescente e juvenil), (com 20 pacientes; 71%), seguido da congênita (4 pacientes; 14%), neuromuscular (3 pacientes; 11%) e diplomielia (1 paciente; 4%) ( Tabela 1 ).

**Tabela 1:** Dados sociodemográficos

Paciente	Etiologia da escoliose	Altura pré procedimento(m)	Peso (Kg)	Sexo
M1	Idiopática do adolescente	1.65	46	Feminino
M2	Neuromuscular	1.43	30	Feminino
M3	Congênita	1.39	35	Feminino
M4	Idiopática do adolescente	1.56	48	Feminino
M5	Idiopática do adolescente	1.54	46	Feminino
M6	Idiopática do adolescente	1.57	44	Feminino
M7	Idiopática infantil	1.48	60	Feminino
M8	Idiopática do adolescente	1.62	43	Feminino
M9	Diplomielia	1.35	28	Feminino
M10	Idiopática do adolescente	1.66	55	Feminino
M11	Idiopática do adolescente	1.55	54	Feminino
M12	Idiopática juvenil	1.72	59	Feminino
M13	Idiopática Infantil	1.59	43	Feminino
M14	Idiopática do Adolescente	1.65	52.5	Feminino
M15	Idiopática do adolescente	1.59	47	Feminino
M16	Neuromuscular	1.30	30	Masculino
M17	Idiopática juvenil	1.42	41	Masculino
M18	Congênita	1.64	60	Masculino
M19	Idiopática juvenil	1.53	37.2	Masculino
M20	Idiopática do adolescente	1.61	47	Feminino
M21	Neuromuscular	1.30	22	Feminino
M22	Congênita	1.61	46	Feminino
M23	Idiopática juvenil	1.68	55	Masculino
M24	Idiopática do adolescente	1.66	50	Feminino
M25	Idiopática do adolescente	1.62	56	Feminino
M26	Congênita	1.27	23	Feminino
M27	Idiopática do adolescente	1.64	46	Feminino
M28	Idiopática do adolescente	1.52	38	Feminino

Nota: Especificação dos tipos de escoliose, altura, peso e sexo.

Fonte: Autores (2023).

A idade média de diagnóstico/indicação de cirurgia foi de 10,2 anos; a idade de cirurgia de 15,1 anos e a média de atraso entre o diagnóstico e realização do procedimento de 4,7 anos.

## Resultados Intraoperatórios

Sobre os dados das cirurgias ( Tabela 2 ), a média de perda sanguínea foi de 768,61 ml, e 3 pacientes necessitaram de transfusão sanguínea. O tempo médio de duração das cirurgias foi 200.74 minutos. Alterações neurofisiológicas transitórias foram identificadas em cinco pacientes, porém não houve déficits neurológicos no pós-operatório.

**Tabela 2:** Dados da cirurgia

Paciente	Perda sanguínea (ml)	Alteração neurofisiológica	Descrição dos episódios	Técnicas	Nível proximal incluído na artrodese	Nível distal incluído na artrodese	Tempo de cirurgia(min)
M1	790	Sim	Queda de potencial motor no momento da derrotação. Sinais normalizados após retirada da barra. Cirurgia finalizada sem mais intercorrências	NA	T8	L3	180
M2	870	Não	NA	Tração intraoperatória: Bipolar	T1	Ílio	310
M3	110	Não	NA	Laminectomia; TLIF	L4	S1	180
M4	580	Não	NA	NA	T4	L2	200
M5	1.115	Não	NA	NA	T4	T12	180
M6	470	Sim	Queda de potencial motor à esquerda. Retorno do potencial após aumento de PAM e temperatura da sala	Tração intraoperatória	T4	L1	220
M7	970	Sim	Queda de Potencial a esquerda que retornou após retirada da tração	Tração intraoperatória;	T2	L2	180
M8	650	Não	NA	NA	T11	L3	100
M9	1.140	Não	NA	Tração intraoperatória; Costoplastia	T3	L3	240
M10	550	Não	NA	Tração intraoperatória	T4	L3	300
M11	960	Não	NA	NA	T4	L4	200
M12	1.400	Não	NA	Osteotomias (3)	T4	L4	300
M13	1.010	Não	NA	Tração intraoperatória	T4	L2	200
M14	610	Não	NA	NA	T4	T12	145
M15	900	Não	NA	NA	T6	L3	135
M16	350	Não	NA	Tração intraoperatória; Bipolar	T1	Ílio	250
M17	1.500	Não	NA	Tração intraoperatória; Osteotomia (3)	T2	L2	220
M18	560	Não	NA	Osteotomias (3)	T2	L2	280
M19	980	Não	NA	Osteotomias (3); Tração intraoperatória	T4	L3	120
M20	690	Não	NA	NA	T10	L4	120
M21	840	Não	NA	Tração intraoperatória; Bipolar	T1	Ílio	220
M22	1.250	Sim	Queda de potencial motor e sensitivo durante osteotomia. Potencial retorna após finalização da descompressão e osteotomia	OSP assimétrico T10	T6	L3	270
M23	740	Não	NA	NA	T3	T12	200
M24	950	Não	NA	Osteotomias(3)	T3	L2	200
M25	300	Não	NA	NA	T4	T12	100
M26	330	Sim	Queda de potencial em MID, retornou após diminuição da tração	Tração intraoperatória	C7	L1	240
M27	820	Não	NA	Translação PL	T4	L3	200
M28	590	Não	NA	NA	T11	L4	150

Abreviaturas: OSP, osteotomia de subtração pedicular; TLIF, técnica de fusão lombar transforaminal.

Nota: Especificação do sangramento intra-operatório, alterações neurofisiológicas, procedimentos realizados, níveis de artrodese e tempo cirúrgico.

Fonte: Autores (2023).

Procedimentos de tração e osteotomias tipo 2 foram realizadas em 17 pacientes; cinco foram submetidos a tração intraoperatória; três foram submetidos a tração intraoperatória combinada à técnica bipolar; três fizeram procedimento de tração intraoperatória e osteotomias e um fez tração intraoperatória combinada à costoplastia; três fizeram apenas osteotomias.

Outros procedimentos realizados nas cirurgias foram: osteostomia de subtração pedicular (OSP) assimétrica de T10; laminectomia; e técnica de fusão lombar transforaminal (TLIF, na sigla em inglês).

Com relação à artrodese, o nível proximal da inserção de parafusos localizou-se na região torácica, em sua maioria na vértebra T4; em 1 paciente ocorreu na região cervical (C7) e em outro, na região lombar (L4). O nível distal concentrou-se na região lombar; em 4 pacientes ocorreu na região torácica (T12), em 1 paciente na região do sacro (S1) e em 3 pacientes, o procedimento foi realizado na região do quadril (ílio).

Todos os pacientes receberam implantes. Foram necessários 457 implantes, entre eles parafusos, hastes, ganchos e bandas sublaminares.

## Resultados Pós-operatórios Clínicos e Complicações

Os dados pós-operatórios clínicos ( Tabela 3 ) mostram que o tempo médio de alta foi de 6.92 dias. Todos os pacientes apresentaram secreções ou sangue no dreno no 1° dia após a cirurgia (média de 370.05 ml), 27 (96%) pacientes no 2° dia (média de 252.59 ml), 9 (32%) no 3° dia (média de 144.66 ml), e apenas um (4%) no 4° dia (280 ml).

**Tabela 3:** Resultados pós-operatório

Paciente	Tempo de alta	Débito dreno D1 (ml)	Débito dreno D2 (ml)	Débito dreno D3 (ml)	Débito dreno D4 (ml)	Bolsas de sangue transfundidas	Quantas	Hemoglobina PO1	Hemoglobina PO3	Infecções	Descrição das infecções	Outras complicações	Descrição das complicações
M1	5 dias	225	175	150		Sim	2	8.2	9,8	não		sim	Náuseas e vômitos
M2	12 dias	680	350			Sim	3	12.6	12.8	não		não	
M3	4 dias	300	200			Não		11.1	11.0	não		não	
M4	4 dias	370	250			Não		10.6	10.1	não		não	
M5	6 dias	317	150			não		10.2	9.9	não		não	
M6	8 dias	454	300			não		9.4	9.1	não		não	
M7	7 dias	144	180			sim	2	8.2	10.2	não		sim	Náuseas e vômitos
M8	5 dias	300	150	86		não		9.0	8.1	não		sim	Náuseas e vômitos
M9	5 dias	480	200			sim	2	8.1	12.2	não		sim	Dor intensa
M10	6 dias	290	278			não		10.1	10.7	não		sim	Dispneias aos médios esforços
M11	6 dias	450	140			sim	4	8.4	12.3	não		sim	Náuseas e vômitos
M12	18 dias	250	500	500		sim	3	7.5	9.3	sim	Infecção de sítio cirúrgico por *Klebsiella pneumoniae*	sim	FO secretiva, desbridamento dia 12/02
M13	7 dias	155	45			não		9.7	8.1	não		sim	Náuseas e vômitos
M14	6 dias	300	188			sim	1	8.5	10.7	não		sim	Dor moderada
M15	5 dias	390	380	120		não		9.5	9.6	não		não	
M16	14 dias	630	450			não		11.2	10.3	sim	ITU por enterobacter cloacae	sim	Úlcera sacral por estase
M17	5 dias	550	190			sim	3	7.6	10.5	não		sim	Acidose metabólica
M18	5 dias	250	450	53		não		13.6	11.3	não		não	
M19	6 dias	500	150			sim	1	9.7	11.0	não		sim	Acidose metabólica
M20	18 dias	370	500	115	280	sim	2	9.1	9.4	não		sim	Obstrução funcional, crises convulsivas, hipocalemia.
M21	8 dias	400	344			não		10.9	11.1	não		não	
M22	5 dias	725	400	118		não		9.0	8.4	não		sim	Dor intensa
M23	5 dias	400	200			não		11.4	11.0	não		não	
M24	6 dias	303	100			não		9.4	8.4	não		sim	Bexigoma, acidose metabolica
M25	4 dias	350	170	100		não		8.9	8.9	não		não	
M26	5 dias	358	180			sim	2	8.0	11.2	não		não	
M27	5 dias	200				sim	2	8.0	10.9	não		sim	Náuseas e vômitos
M28	4 dias	220	200	60		não		10.3	10.4	não		não	

Abreviaturas: FO, ferida operatória; ITU, infecção do trato urinário; M, masculino.

Nota: Especificação de variáveis pós-operatórias e complicações.

Fonte: Autores (2023).

Dos pacientes, 12 (43%) precisaram receber bolsas de sangue no pós-operatório. Seis receberam duas; três receberam três, dois receberam uma e um recebeu quatro bolsas.

Dentre as complicações observadas, houve infecção em dois pacientes, uma urinária com a bactéria *Enterobacter cloacae* e outra de sítio cirúrgico por *Klebsiella pneumoniae* . Analisando em termos percentuais, por etiologia, obtivemos 5% de infecções de sítio operatório em casos idiopáticos, sem outras infecções de sítio operatório. Dezesseis pacientes (57%) apresentaram alguma intercorrência; oito apresentaram náuseas e vômitos; dois tiveram dor intensa e um apresentou dor moderada; dois pacientes tiveram acidose metabólica, um apresentou acidose e alterações urinárias (bexigoma); um teve úlcera sacral por estase; um precisou passar por desbridamento cirúrgico, um paciente apresentou dispneias aos médios esforços e um apresentou obstrução funcional, crises convulsivas e hipocalemia.

## Resultados Radiográficos Pré e Pós-operatórios

Quanto aos dados radiográficos, observou-se uma média pré-operatória do ângulo de Cobb da curva torácica alta de 29,3 graus (0–68), torácica baixa de 63,6 graus (0–120), lombar de 42,2 graus (16–79), cifose torácica de 36,6 graus (5–75) e lordose lombar de 55,6 graus (8–86) ( Tabela 4 ).

**Tabela 4:** Resultados radiográficos

Paciente	Cobb TP pre (graus)	Cobb TD pré-operatório (graus)	Cobb lombar pré-operatório (graus)	Cobb cifose Pré-operatório (graus)	Cobb lordose pré-operatório (graus)	Cobb TP pós-operatório (graus)	Cobb TD pós-operatório (graus)	Cobb lombar pós-operatório (graus)	Cobb cifose pós-operatório (graus)	Cobb lordose pós-operatório (graus)
M1	22	49	35	43	60	20	29	21	2	36
M2	50	86	36	17	46	34	61	25	4	56
M3	0	0	55	38	66	0	0	50	38	46
M4	35	72	38	16	58	16	23	9	27	47
M5	30	48	36	27	57	3	27	31	20	50
M6	33	106	55	47	50	22	29	34	36	45
M7	49	83	44	58	86	38	44	25	49	68
M8	15	36	45	5	30	24	33	16	13	34
M9	68	120	20	75	56	20	56	0	40	52
M10	27	56	68	5	48	5	23	48	33	45
M11	66	103	40	41	55	56	54	21	15	49
M12	0	50	79	5	8	0	26	23	19	33
M13	49	78	43	28	57	35	44	20	39	55
M14	9	54	40	18	65	15	25	7	32	20
M15	16	49	48	38	71	15	37	31	30	52
M16	12	22	50	28	72	20	14	34	28	80
M17	30	97	37	70	45	27	65	25	50	50
M18	40	74	47	56	58	33	54	27	41	36
M19	30	94	38	58	53	35	38	17	38	40
M20	6	36	47	36	52	6	10	20	40	50
M21	45	101	16	62	68	40	78	2	3	45
M22	51	89	44	70	76	28	53	21	55	55
M23	23	60	34	25	43	26	30	26	28	35
M24	43	57	30	42	56	29	35	16	38	50
M25	19	40	20	10	71	5	0	0	12	60
M26	45	87	31	65	57	37	48	10	33	24
M27	6	0	40	23	48	0	0	16	40	40
M28	2	34	66	19	45	0	24	27	30	37
	29,32142857	63,60714286	42,21428571	36,60714286	55,60714286	21,03571429	34,28571429	21,5	29,75	46,55555556
Percentual de correção das curvas			TA: 28%	TB: 47%	L: 50%					

Abreviaturas: L, lombar; M, masculine; TA, torácica alta; TB, torácica baixa.

Nota: Resultados radiográficos calculados pelo método de Cobb pré e pós-operatórios.

Fonte: Autores (2023).

No pós-cirúrgico imediato, conseguiu-se uma correção em média de 28% da curva torácica alta, 47% da curva torácica baixa e 50% da curva lombar (médias pós-operatórias de 21 graus, 34,2 graus e 21,5 graus, respectivamente).

Abordando separadamente as escolioses não-idiopáticas, obtivemos, em suas curvas principais, percentual de correção de 28% nas neuromusculares e 30% nas congênitas.

## Resultados organizacionais e de logística

Sobre a estrutura hospitalar, contou-se com ambulatórios, acesso ao setor de radiologia, 5 salas cirúrgicas exclusivas disponíveis durante 4 dias e cerca de 10 a 15 vagas de UTI para rotina pós-operatória. A equipe cirúrgica contou com médicos voluntários e com médicos do quadro de funcionários do próprio hospital. Os implantes foram obtidos por doação, não sendo possível então, avaliar os custos envolvidos com precisão.

As cirurgias aconteceram em fevereiro de 2021, em um período de 4 dias, contudo, a organização do mutirão se iniciou em 2020 ( Fig. 1 ).

**Fig. 1:** Cronograma de execução do mutirão.

## Discussão

Observou-se uma prevalência de pacientes do sexo feminino, 24 (86%) dentre os 28 pacientes, coincidindo com outro estudo no qual participaram 169 pacientes, 121 (71,6%) dos quais eram do sexo feminino. 7 Outro estudo sobre a incidência da escoliose idiopática do adolescente (EIA) em diversos países, notou que a prevalência e a gravidade da escoliose são maiores em meninas. 8

Esta pesquisa também constatou uma maior prevalência da escoliose idiopática, contabilizando 20 casos (71%). A EIA é uma doença com uma prevalência geral de 0,47 a 5,2% na literatura atual. 8 Na revisão realizada por Pereira e Gomes, 9 menciona-se estudos que corroboram os resultados desta pesquisa: amostra de 358 adolescentes, 16 com EIA, prevalência de 4,8% 10 ; amostra de 3.105 adolescentes, 38 com EIA, prevalência em 5,3% 11 ; 418 indivíduos, 18 adolescentes com EIA, prevalência de 4,3% 12 ; e 2.562 adolescentes, 37 com EIA, prevalência de 1,5%. 13 Tais dados demonstram que nosso estudo se encontra em consonância com a literatura no aspecto epidemiológico.

Em relação a complicações pós-operatórias, desconfortos são considerados comuns, dentre eles náuseas e vômitos nas primeiras horas de pós-operatório devido à realimentação oral ou em razão da anestesia, dispneia, oligúria, 14 odinofagia, dor nos locais das injeções intravenosas, insônia e constipação. 15 16 Observou-se que oito pacientes apresentaram náuseas e vômitos, um apresentou dispneias aos médios esforços e um apresentou obstrução funcional, reações comuns em pacientes no pós-operatório.

Com relação à dor, ressalta-se que “[...] a ferida cirúrgica não é espontaneamente dolorosa após 48 horas do ato cirúrgico 14 ”; desta forma, é importante averiguar o nível de dor do paciente e realizar os procedimentos necessários. Dentre os 28 pacientes, apenas 2 apresentaram dor intensa e 1 apresentou dor moderada, sem outras complicações.

Outras ocorrências mais dolorosas podem acontecer no pós-operatório de procedimentos cirúrgicos em geral: sangramento, infecção da ferida, trombose venosa, insuficiência respiratória, tromboembolismo pulmonar, atelectasia pulmonar e infecção urinária. 15 16

Outros estudos sobre infecções no pós-cirúrgico de pacientes que se submeteram a tratamento de deformidades na coluna vertebral relatam infecções no trato urinário, perda de controle esfincteriano, contaminação, infecções de feridas ou distúrbios gastrointestinais e complicações pulmonares, 17 além de maior risco de infecções em pacientes com escoliose neuromuscular do que aqueles com EIA. Neste estudo, as complicações e infecções relatadas são comuns em pacientes no pós-operatório de uma forma geral. 18

A perda de sensibilidade nas extremidades pode ocasionar perda de controle do intestino ou da bexiga e está mais associada a pacientes com escoliose neuromuscular. 18 As complicações observadas no paciente M16 (neuromuscular) podem estar relacionadas com o tipo de escoliose; o paciente não deambulava e apresentou infecção urinária e úlcera sacral.

A complicação mais grave foi apresentada pelo paciente M12 (EIA), que evoluiu com infecção da ferida operatória, uma complicação também observada em outros estudos. 15 16 17 18 O tratamento utilizado foi a desbridamento cirúrgico, mencionado como tratamento usual, 18 juntamente com a irrigação do sítio cirúrgico. Na literatura, a taxa de infecção em cirurgia para EIA variou de 0,9 a 3%; já para a escoliose neuromuscular, a taxa de infecção variou de 4,2 a 20%. Neste estudo, a prevalência de infecção da ferida operatória ficou aproximadamente em 3,5% na análise geral e de 5% entre os casos idiopáticos.

Quanto a outras complicações, os dados irão depender das bases de dados consultadas, variando de 5 a 23% em EIA. Dados mais recentes do banco de dados da SRS, de 2011, revelaram taxa de complicação de 6,3% para todos os casos de EI. Neste estudo os dados mostram que 16 pacientes (57%) apresentaram complicações, aquelas associadas ao tipo específico de cirurgia acometeram 6 dos 28 pacientes, com taxa de prevalência de 21%, sendo apenas uma complicação mais séria (que gerou reoperação), a infecção do sítio cirúrgico.

As taxas de complicações e infecções coincidem com o que é observado na literatura; desta forma, o tempo curto da realização das cirurgias não está relacionado com as complicações observadas.

Com relação ao aspecto radiográfico, houve uma correção média geral de 28% da curva torácica alta, 47% da curva torácica baixa e 50% da curva lombar (médias pós-operatórias de 21 graus, 34,2 graus e 21,5 graus). Estratificando pelas etiologias não-idiopáticas, em suas curvas principais, obtivemos correção de 28% nas neuromusculares e 30% nas congênitas. Em um estudo 19 que analisou vários resultados pós-cirúrgicos, um artigo publicado em 1973, com 71 participantes utilizando hastes de Harrington, gesso de Risser e deambulação precoce, relatou que a curva média pré-operatória era de 56°, com correção de 54% no dia da cirurgia e uma correção de 46% no seguimento. Em 1989 (352 participantes), em pacientes tratados por fusão posterior da coluna, a curva pré-operatória média foi de 54° e a correção média de inclinação ativa supina pré-operatória de 48%. A correção média na cirurgia foi 52% e no seguimento de 2 anos 40%. 19

Em 2004, um estudo comparativo de 4 diferentes instrumentações (haste dupla, multissistemas de gancho), que avaliou 127 pacientes, com o uso dos sistemas C-D Horizon, Moss-Miami, TSRH e Isola, sendo a correção média de 63% para o C-D Horizon e Moss-Miami, e 58% para TSRH e Isola. 19

As correções das curvas ao longo dos anos permaneceram similares, e os valores alcançados nessa pesquisa são semelhantes aos outros estudos, confirmando o fato de que apesar de não corrigir completamente a curva, obteve-se um resultado satisfatório. 19 As Figs. 2 3 4 5 demonstram de forma visual o nível de correção alcançado em alguns pacientes.

**Fig. 2:** Paciente M21 pré-operatório. Fonte: Autores (2023).

**Fig. 3:** Paciente M1 resultados pós-cirúrgicos. Fonte: Autores (2023).

**Fig. 4:** Paciente M16 pré-cirúrgico. Fonte: Autores (2023).

**Fig. 5:** Paciente M16 resultados pós-cirúrgico. Fonte: Autores (2023).

Com relação a questão logística, algumas dificuldades foram observadas na realização do mutirão, mas não termos trabalhos similares que abordaram tal aspecto. Observamos que no período pré-operatório, houve dificuldade na divulgação do ambulatório de triagem e em estabelecer contato com os diversos pacientes; além disso, houve a necessidade de técnicos de radiologia capacitados para a realização de radiografias panorâmicas da coluna em ortostase. Observamos, também, que houve a necessidade de salas de atendimento amplas para confecção de fotos e avaliação clínica dos pacientes no ambulatório de triagem. Nos aspectos da avaliação pré-operatória, também houve a necessidade de ambulatório de anestesiologia para avaliação e preparação dos pacientes para o procedimento.

As dificuldades logísticas no intraoperatório dizem respeito a reunir equipe e recursos no período estipulado para o mutirão. Para a sua realização foi necessário reunir uma equipe de profissionais de várias áreas de atuação e de estados diferentes, com experiência em cirurgia de escoliose que estivessem disponíveis no período de realização do mutirão.

No pós-operatório, as dificuldades encontradas foram na disponibilização de salas de UTI. Eram necessários cerca de 10 a 15 leitos simultaneamente, pelo fato de, por vezes, pacientes mais graves não conseguirem receber alta da UTI no primeiro dia pós-operatório. Além disso, era necessário equipe de enfermagem e fisioterapia treinada em procedimentos de tratamento de escoliose, para manutenção de drenos, trocas de curativo ou por conta de deambulação precoce.

Os artigos estudados que tratavam da realização de mutirão 5 7 para correção de curva de escoliose não trouxeram dados acerca das dificuldades logísticas; tampouco falaram sobre complicações apresentadas pelos pacientes que pudessem justificar a não realização de mutirões de cirurgia. Contudo, algumas reportagens forneceram dados acerca de mutirões cirúrgicos e ressaltam pontos importantes a serem observados.

O Conselho Regional de Medicina do Estado da Bahia (CREMEB) 20 lançou nota em 2018, alertando para algumas questões relacionadas a mutirão de cirurgias, pois alguns problemas podem surgir devido às condições em que essas cirurgias venham a ser oferecidas, além dos riscos de complicações nos pacientes submetidos aos processos cirúrgicos, especialmente por nem sempre ter pessoal qualificado para acompanhamento durante e após a cirurgia.

Apesar das reportagens acerca de complicações relacionadas a mutirões, nenhuma trata de cirurgia de escoliose, e os casos que apresentaram problemas são baixos se comparados à quantidade de pessoas que são beneficiadas.

Em dezembro de 2022, o Centro Estadual de Reabilitação e Readaptação Dr. Henrique Santillo (CRER), um centro de reabilitação da Secretaria de Saúde do Governo de Goiás (SES-GO), realizou mutirão de cirurgias eletivas de escoliose para pacientes na lista do SUS. Foram atendidos 20 pacientes, alguns dos quais já aguardavam há cerca de 5 anos, que graças ao mutirão tiveram a oportunidade de melhorar sua qualidade de vida. 21

Em Pernambuco, a equipe de traumatologia e ortopedia do Hospital Otávio de Freitas realizou um mutirão cirúrgico para tratamento de escoliose e atendeu 18 pacientes com o objetivo de minimizar a fila do SUS. Foram realizados 4 procedimentos por dia, em 3 salas cirúrgicas separadas para essa ação e utilizados 16 leitos de enfermaria adulta e pediátrica, bloco cirúrgico de trauma, UTI e sala de recuperação. 22

Nesta pesquisa, a idade média de diagnóstico foi de 10,2 anos e a de cirurgia 15,1 anos, sendo o tempo de espera para o procedimento de 4,7 anos, no Brasil, conforme estudo 23 realizado com 51 pacientes todos receberam diagnóstico com idades entre 10 e 17 anos, e a média de espera pela cirurgia foi de 25,41 meses (variando entre 2–180 meses), alguns pacientes esperaram por até 15 anos. Esse tempo de espera pode comprometer a qualidade de vida do paciente, afetando sua autoimagem, satisfação e funcionalidade. 24

No presente estudo, não analisamos questionários de qualidade de vida e satisfação pessoal devido a dificuldades logísticas.

## Conclusão

Apesar das dificuldades identificadas para a organização de ações similares e de algumas complicações observadas, parece ser viável estimular a multiplicação desse evento em um maior número de hospitais, tendo em vista o número elevado de pacientes em filas de espera de cirurgias aguardando tratamento cirúrgico para escoliose. Porém, é importante salientar que necessitamos de mais ações nesse modelo para avaliação mais acurada de sua segurança e aplicabilidade, principalmente no que diz respeito a casos mais graves e não-idiopáticos.
